# Supplementary material for: Feature-space selection with banded ridge regression
Source: Neuroimage. Author manuscript; Available in PMC 2023 Jan 2. (PMC9807218; doi:10.1016/j.neuroimage.2022.119728)
Supplement: 1 [file NIHMS1860175-supplement-1.pdf]

Feature-space selection with banded ridge regression

Tom Dupré la Tour[1], Michael Eickenberg[1,2], Anwar O. Nunez-Elizalde[1], and Jack L. Gallant[1,3,*]

[1]Helen Wills Neuroscience Institute, University of California, Berkeley, CA 94720, USA
[2]Center for Computational Mathematics, Flatiron Institute, 162 5th Ave, New York, NY 10100, USA
[3]Department of Psychology, University of California, Berkeley, CA 94720, USA
[*]corresponding author: gallant@berkeley.edu

A Supplementary materials

A.1 Quantifying feature-space selection with the effective rank

In Sections 3.4 and 3.7, two examples are proposed to demonstrate the feature-space selection mechanism in banded ridge regression. For the sake of demonstration, these examples use the *effective rank* to quantify the number of feature spaces effectively used in the model. This section explains why the effective rank metric was chosen, and compares alternative definitions of the effective rank.

To quantify the number of feature spaces effectively used in the model, one candidate metric is to count the number of feature spaces with a non-zero effect on the prediction. However, because the hyperparameters optimized by banded ridge regression never reach infinity, the coefficients b^* are never exactly zero, even for feature spaces that are effectively ignored. For this reason, all feature spaces have non-zero values in the variance decomposition, and one cannot simply count the number of non-zero values. To fix this issue, it is possible to define a positive threshold, and to count the number of values above the threshold. Yet, this threshold would have an arbitrary value, and would artificially separate values that are slightly above from values that are slightly below, leading to a discontinuity in the metric. Instead, a better metric of the number of feature spaces effectively used in the model would vary continuously as some feature space contribution goes to zero.

To address this issue, we leverage a metric to compute the rank of a linear system. The rank of a linear system is the number of independent equations in the system, and corresponds to the number of non-zero eigenvalues λ_i. The rank is thus a discontinuous function of the eigenvalues. To remove the discontinuity, the rank can be approximated by the *effective rank*. Several effective rank definitions exist, such as e [Roy and Vetterli, 2007], r_0 [Bartlett et al., 2020], R_0 [Langeberg et al., 2019, Bartlett et al., 2020], and h (proposed in this work). Assuming the eigenvalues are non-negative and sorted in decreasing order ($\lambda_0 \geq \lambda_1 \geq \ldots \geq \lambda_{m-1} \geq 0$), these four metrics are defined as

$$e = \exp(\sum_{i=0}^{m-1} \tilde{\lambda}_i \log(\tilde{\lambda}_i)), \qquad \text{with} \qquad \tilde{\lambda}_i = \frac{\lambda_i}{\sum_{i=0}^{m-1} \lambda_i}, \tag{1}$$

$$r_0 = \frac{\sum_{i=0}^{m-1} \lambda_i}{\lambda_0}, \qquad R_0 = \frac{(\sum_{i=0}^{m-1} \lambda_i)^2}{\sum_{i=0}^{m-1} \lambda_i^2}, \qquad h = 1 + 2\frac{\sum_{i=0}^{m-1} i\lambda_i}{\sum_{i=0}^{m-1} \lambda_i}. \tag{2}$$

All four metrics (e, r_0, R_0, and h) have similar useful properties. They are continuous functions of the m eigenvalues, with values in the interval $[1, m]$. Because they are continuous, they do not require defining any threshold. They are also not affected by additional unused dimensions (dimensions i with $\lambda_i = 0$). They are equal to k when k eigenvalues are equal (and non-zero) and all the other eigenvalues are equal to zero. The four metrics only differ in the way these integer values are interpolated. To describe how they interpolate these integer values, Figure 1 presents the metric values on different 2D and 3D spaces.

To use an effective rank metric to quantify the number of feature spaces effectively used in the model, the following procedure is used. First, the variance decomposition $\rho \in \mathbb{R}^m$ is computed, for example using the product measure as described in Section 2.6. Then, negative values are clipped to zero, and an effective rank metric is computed on the vector ρ. Although all four metrics give similar results, the definition e was used throughout this paper for its intuitive link to the entropy $H = -\sum_i \lambda_i \log(\lambda_i)$ [Roy and Vetterli, 2007, Araki and Lieb, 2002, Bach, 2022]. Because the effective rank estimates the number of feature spaces effectively used over m features spaces, it is noted $\tilde{m}$.

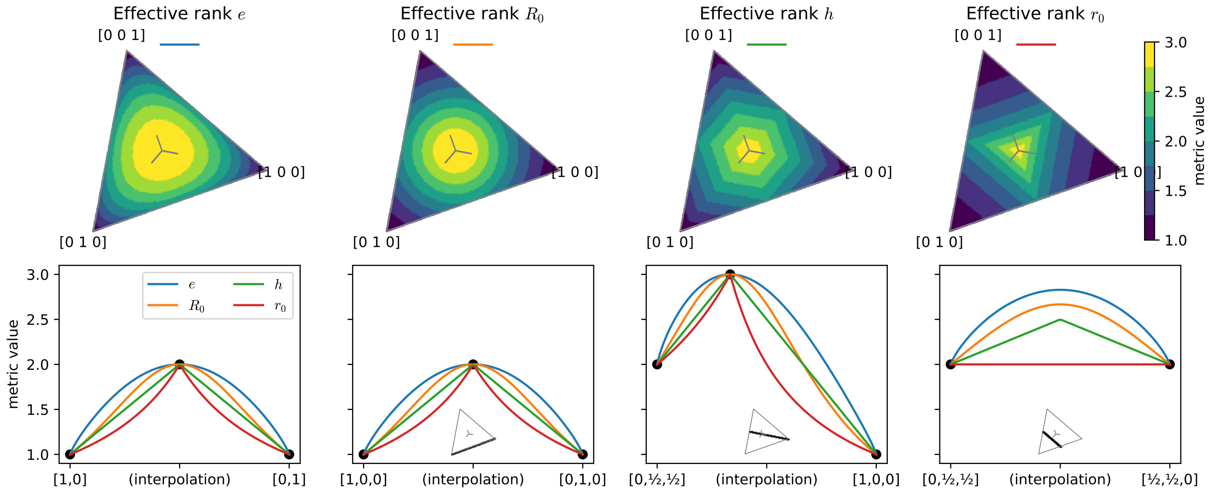

Figure 1: Comparison of four effective rank metrics. Effective rank metrics are useful to quantify the number of feature spaces used in banded ridge regression. **(Top)** Values of the four metrics on the simplex. The variance decompositions are defined on the simplex Δ_{m-1}, the space of vectors in dimension m with positive values that sum to one. With $m = 3$, the simplex is a 2-dimensional surface in the shape of a triangle. All four effective rank metrics are equal to 1 at the triangle corners, to 2 at the edge midpoints, and to 3 at the center. The four metrics differ in the way they interpolate these integer values. The color mapping is discrete to highlight the structures of the different metrics. **(Bottom)** Values of the four metrics on different 1-dimensional trajectories, linearly interpolating between two points in dimension 2 or 3. All four metrics are continuous, and equal to k when the values are equally split over k dimensions (depicted by the black dots). Moreover, adding unused dimensions (second versus first panel) does not alter the metric values. All four metrics are thus reasonable choices for quantifying the number of feature spaces used in banded ridge regression. The definition e was used throughout this paper for its intuitive link to entropy.

A.2 List of the 22 feature spaces from the short-films dataset

In Sections 3.6 and 4.8, banded ridge regression is fit on 22 features spaces from the "short-films" dataset [Nunez-Elizalde et al., 2018]. Here, a short description of the 22 feature spaces is given.

- Stimulus motion energy: spatiotemporal Gabor filters computed on the stimulus luminosity (11,845 features; [Nishimoto et al., 2011]).
- Retinotopic motion energy: spatiotemporal Gabor filters, computed on the stimulus luminosity, corrected for eye movements (11,845 features).
- Eye position: cubic polynomial expansion of the eye position during movie watching (36 features).
- Visual semantic: semantic labels of objects and actions present in the scene [Huth et al., 2012], projected in a semantic space (985 features; [Huth et al., 2016]).
- Visual thematics (5 feature spaces): categorization of verb classes into categories based on the type of events described. VerbNet (85 features; [Kipper et al., 2006]) was used to construct a thematic roles

feature space (36 features), a selective restrictions feature space (38 features) and their interaction (71 features), and a verb categories feature space (101 features).

- Spectrogram: spectral power density of the sound wave (2388 features).
- Wavenet: Auditory features related to musical instruments extracted using a pretrained convolutional neural network (512 features; [Oord et al., 2016]).
- Word rate: cubic polynomial expansion of word rate (13 features).
- Speech syntax position: part-of-speech tags of each word in the sentences, extracted using a pre-trained neural network (12 features; [Andor et al., 2016]).
- Speech syntax labels: word dependencies in the sentence, extracted using a pre-trained neural network (43 features; [Andor et al., 2016]).
- Speech semantic: spoken words projected in a semantic space (985 features; [Huth et al., 2016]).
- Speech thematics (7 feature spaces): categorization of verb classes into categories based on the type of events described. VerbNet [Kipper et al., 2006] labels were used to construct a thematic role feature space (39 features), a verb class feature space (274 features), and a verb category feature space (102 features). Four thematic roles (verb, actor, recipient, place) were further decomposed by projecting words in a semantic space (985 feature each).

A.3 Banded ridge regression is equivalent to multiple-kernel ridge regression

As stated in Section 3.8, banded ridge regression is equivalent to multiple-kernel ridge regression, up to a subtle difference. This subsection first demonstrates the equivalence, then discusses the source and implications of the difference. Note that a deep understanding of the difference is not critical to the rest of the paper.

Equivalence. To reformulate banded ridge regression as a multiple-kernel ridge regression, one can note that banded ridge regression is equivalent to a ridge regression with weighted feature spaces $X_i = X_i * \sqrt{\mu/\lambda_i}$ (see Section 4.3). This ridge regression is in turn equivalent to a kernel ridge regression [Saunders et al., 1998] (see Section 4.2) with a linear kernel $K = \sum_i X_i X_i^\top = \sum_i X_i X_i^\top (\mu/\lambda_i)$. Writing $K_i = X_i X_i^\top$ and $\gamma_i = \mu/\lambda_i$, the model becomes a multiple-kernel ridge regression

$$w^* = \underset{w}{\operatorname{argmin}} \, || \sum_i \gamma_i K_i w y||_2^2 + \mu w^\top \sum_i \gamma_i K_i w. \qquad (3)$$

Multiple-kernel ridge regression corresponds to a kernel ridge regression with a weighted kernel $K = \sum_i \gamma_i K_i$ and a regularization hyperparameter μ. Because μ is arbitrary in the change of variables, one can choose it such that $\sum_i \gamma_i = \sum_i \mu/\lambda_i = 1$, which leads to $\mu = (\sum_i 1/\lambda_i)^{-1}$. In this way, γ is restricted to a subspace called the simplex $\Delta_{m-1} = \{\gamma \in \mathbb{R}_+^m; \sum_i \gamma_i = 1\}$, which corresponds to the standard formulation in the multiple-kernel learning literature [Gönen and Alpaydın, 2011]. Banded ridge regression is thus equivalent to multiple-kernel ridge regression. In Sections 4.5 and 4.6, this equivalence is used to solve banded ridge regression efficiently when the number of features is larger than the number of samples.

Key difference. The equivalence between banded ridge regression and multiple-kernel ridge regression hides a subtle difference in the way hyperparameters are optimized. In banded ridge regression, all hyperparameters $\lambda \in \mathbb{R}_+^m$ are optimized through cross-validation. Thus, in the equivalent multiple-kernel ridge regression, both the kernel weights $\gamma \in \Delta_{m-1}$ and the regularization $\mu > 0$ must be optimized through cross-validation. On the contrary, in the multiple-kernel learning literature, the kernel weights γ are usually learned within the training set [Gönen and Alpaydın, 2011], and only μ is learned through cross-validation. Some rare papers have proposed learning kernel weights through cross-validation [Tsuda et al., 2004, Keerthi et al., 2007, Klatzer and Pock, 2015], but this practice is not common in the multiple-kernel learning literature. This difference is important, and is further discussed in the rest of the subsection. To highlight the difference between the two strategies, the following notations is used:

- **MKRR-cv** is a multiple-kernel ridge regression with kernel weights learned over **c**ross-**v**alidation.
- **MKRR-in** is a multiple-kernel ridge regression with kernel weights learned with**in** the training set.

Using these notations, banded ridge regression is equivalent to MKRR-cv, whereas the standard formulation in multiple kernel learning is MKRR-in. Additionally, the squared group lasso is equivalent to MKRR-in [Bach, 2008, Rakotomamonjy et al., 2008].

Group scalings. To understand the difference between MKRR-cv and MKRR-in, it is useful to consider the concept of group scalings. When feature spaces are unbalanced (for example in terms of number of features), the regularization can have a different strength on each feature space. To fix this issue, multiple studies have proposed that the regularization strength on each feature space could be evened out by adding group scalings to the regularization. In the group lasso literature, group scalings are positive weights d_i defined for each feature space i, which creates a scaled $L_{2,1}$ norm $\sum_i d_i||b_i||_2$. Similarly, in the multiple kernel learning literature, group scalings modify the optimization by constraining kernel weights to a scaled simplex $\tilde{\Delta}_{m-1} = \{\gamma \in \mathbb{R}_+^m; \sum_i \gamma_i d_i^2 = 1\}$ [Bach, 2008]. Note that group scalings are defined additionally to the kernel weights γ_i.

Because feature spaces can be unbalanced in different ways, many group scalings have been proposed in the group lasso and multiple-kernel learning literature: the square root of the number of features [Yuan and Lin, 2006], the kernel trace [Lanckriet et al., 2004], the square root of the kernel trace [Bach et al., 2004], the kernel empirical rank [Bach et al., 2005], or more sophisticated group scalings (*e.g.* [Bach, 2008, Obozinski et al., 2011, Pan and Zhao, 2016]). However, no group scaling emerged as being superior to the others. In MKRR-in, it is thus necessary to test different group scalings, and select the best one through cross-validation. On the contrary, in MKRR-cv the kernel weights $\gamma \in \Delta_{m-1}$ are learned by cross-validation. Thus, group scalings would have no effect on MKRR-cv. In other words, MKRR-cv is equivalent to using MKRR-in with group scalings optimized via cross-validation.

Summary. To summarize this subsection, MKRR-cv and MKRR-in are two variants of multiple-kernel ridge regression. In MKRR-cv, the kernel weights are learned using cross-validation, whereas in MKRR-in, the kernel weights are learned within the training set. Banded ridge regression is equivalent to MKRR-cv, whereas the squared group lasso is equivalent to MKRR-in. In MKRR-in, one can add group scalings to help balance the feature spaces, but the choice of group scaling needs to be cross-validated. In MKRR-cv, group scalings are less important because optimal kernel weights are already learned through cross-validation.

A.4 Derivation of the hyperparameter gradient

In Section 4.6, hyperparameter gradient descent is used to optimize hyperparameters in multiple-kernel ridge regression. Hyperparameter gradient descent is a method that iteratively improves hyperparameters. At each iteration, the update is based on the gradient of the cross-validation loss L_{val} with respect to hyperparameters δ. Here, the computation of this gradient is derived using the implicit differention framework.

As explained in Section 4.6, multiple-kernel ridge regression is reparameterized with hyperparameters $\delta \in \mathbb{R}^m$. The training and validation loss functions are defined as

$$\mathcal{L}_{\text{train}}(w, \delta) = \left\| \sum_{i=1}^{m} e^{\delta_i} K_{\text{train},i} w - y_{\text{train}} \right\|_2^2 + w^\top \sum_{i=1}^{m} e^{\delta_i} K_{\text{train},i} w, \tag{4}$$

$$\mathcal{L}_{\text{val}}(w^*(\delta), \delta) = \left\| \sum_{i=1}^{m} e^{\delta_i} K_{\text{val},i} w^*(\delta) - y_{\text{val}} \right\|_2^2, \tag{5}$$

where $w^*(\delta) = \text{argmin}_w \mathcal{L}_{\text{train}}(w, \delta)$, and for each feature space i, $K_{\text{train}}, i = X_{\text{train}}, iX_{\text{train}}^\top, i$ is the training kernel, and $K_{\text{val}}, i = X_{\text{val}}, iX_{\text{train}}^\top, i$ is the validation cross-kernel.

Implicit differentiation framework. At each iteration, gradient descent updates the hyperparameters δ in the direction of the gradient of the cross-validation loss. The cross-validation loss $\mathcal{L}_{\text{val}}(w^*(\delta), \delta)$ has two dependencies in δ, one *direct*, and one *indirect* through the dual weights $w^*(\delta)$. The gradient of $\mathcal{L}_{\text{val}}$ with respect to δ is thus the sum of the direct gradient $\frac{\partial \mathcal{L}_{\text{val}}}{\partial \delta}$, and the indirect gradient $\frac{\partial \mathcal{L}_{\text{val}}}{\partial w^*} \frac{\partial w^*}{\partial \delta}$. In the indirect gradient, the Jacobian $\frac{\partial w^*}{\partial \delta}$ can be computed with *implicit differentiation* [Larsen et al., 1996, Chapelle et al.,

2002, Foo et al., 2007], which leads to the full gradient expression

$$\frac{\partial \mathcal{L}^*_{\text{val}}}{\partial \delta} = \frac{\partial \mathcal{L}_{\text{val}}}{\partial \delta} - \frac{\partial \mathcal{L}_{\text{val}}}{\partial w^*} \left(\frac{\partial^2 \mathcal{L}_{\text{train}}}{\partial w \partial w^\top} \right)^{-1} \frac{\partial^2 \mathcal{L}_{\text{train}}}{\partial w \partial \delta^\top}. \tag{6}$$

In this expression, the most expensive term to compute is the inverse Hessian $(\frac{\partial^2 \mathcal{L}_{\text{train}}}{\partial w \partial w^\top})^{-1}$. To reduce this computational bottleneck, three separate approximations are considered. The first approximation, proposed in [Pedregosa, 2016], uses a conjugate gradient method to solve $\frac{\partial \mathcal{L}_{\text{val}}}{\partial w^*}(\frac{\partial^2 \mathcal{L}_{\text{train}}}{\partial w \partial w^\top})^{-1}$ up to a limited precision. The second approximation, proposed in [Lorraine et al., 2019], uses Neumann series to estimate the inverse Hessian with $H^{-1} \approx \sum_{j=0}^{k} (I_n - H)^j$, with typically $k = 5$. The third approximation is more aggressive, and approximates the full gradient with the direct gradient $\frac{\partial \mathcal{L}_{\text{val}}}{\partial \delta}$. This last approximation is equivalent to assuming the inverse Hessian to be 0. In many applications, this approximation is not available because the direct gradient is equal to zero [Lorraine et al., 2019]. However, the direct gradient is not equal to zero in multiple-kernel ridge regression, so it can be used to approximate the full gradient.

Application to multiple-kernel ridge regression. The implicit differentiation framework can be immediately applied to multiple-kernel ridge regression. Yet, some improvements can be done to maximize efficiency. For example, the gradient of the training loss at optimum $\frac{\partial \mathcal{L}_{\text{train}}}{\partial w}\big|_{w^*} = K_{\text{train}}((K_{\text{train}} + I_n)w^* - y_{\text{train}}) = 0$ can be simplified to $(K_{\text{train}} + I_n)w^* - y_{\text{train}} = 0$ by using the gradient with respect to the scalar product $\langle x, y \rangle_K = x^\top K y$. This change improves gradient conditioning, and leads to the same solutions when the kernel has full rank. In addition, this change largely simplifies the following derivative expressions and decreases their computational cost

$$\frac{\partial \mathcal{L}_{\text{train}}}{\partial w} = (K_{\text{train}} + I_n)w - y_{\text{train}}, \qquad \frac{\partial^2 \mathcal{L}_{\text{train}}}{\partial w \partial w^\top} = K_{\text{train}} + I_n, \qquad \frac{\partial^2 \mathcal{L}_{\text{train}}}{\partial w \partial \delta_i} = e^{\delta_i} K_{\text{train},i} w. \tag{7}$$

The other derivative expressions are not affected by this change, and are equal to

$$\frac{\partial \mathcal{L}_{\text{val}}}{\partial w} = K_{\text{val}}(K_{\text{val}} w - y_{\text{val}}), \qquad \frac{\partial \mathcal{L}_{\text{val}}}{\partial \delta_i} = e^{\delta_i}(K_{\text{val}} w - y_{\text{val}})(K_{\text{val},i} w)^\top. \tag{8}$$

Once the gradient $\frac{\partial \mathcal{L}^*_{\text{val}}}{\partial \delta}$ is computed, gradient descent improves the hyperparameters δ with the update $\delta \leftarrow \delta - \eta \frac{\partial \mathcal{L}^*_{\text{val}}}{\partial \delta}$, where η is called the step size. To estimate the optimal step size, one could use the Lipschitz constant of $\frac{\partial \mathcal{L}^*_{\text{val}}}{\partial \delta}$, but that is hard to estimate [Pedregosa, 2016]. Fortunately, the Lipschitz constant of the direct gradient $\frac{\partial \mathcal{L}_{\text{val}}}{\partial \delta}$ can be estimated. Noting $\chi_i = e^{\delta_i} K_{\text{val},i} w$, the validation loss reads $\mathcal{L}_{\text{val}} = ||\sum_{i=1}^m \chi_i - y_{\text{val}}||_2^2$. Using $\frac{\partial \chi_i}{\partial \delta_i} = \chi_i$, the gradient and Hessian are derived as

$$\frac{\partial \mathcal{L}_{\text{val}}}{\partial \delta_i} = \chi_i^\top (\sum_{j=1}^m \chi_j - y_{\text{val}}), \qquad \frac{\partial^2 \mathcal{L}_{\text{val}}}{\partial \delta_i \partial \delta_j} = \chi_i^\top \chi_j + \chi_i^\top (\sum_{k=1}^m \chi_k - y_{\text{val}})[i = j], \tag{9}$$

where $[i = j]$ is equal to 1 if $i = j$ and 0 otherwise. The local Lipschitz constant L is given by the maximum eigenvalue of the Hessian, which can be estimated with power iteration. In our experiments, a step-size $\eta = 1/L$ was used, not only for the direct gradient, but also for the full gradient approximations. Several adaptive step-size strategies were also explored, but none were computationally as efficient as using the estimated Lipschitz constant of the direct gradient. The entire algorithm is listed in pseudo-code in Appendix A.7.

A.5 Hyperparameter Bayesian search

In Section 4.5 and 4.6, two methods are proposed to solved banded ridge regression. Another method proposed in the past to solve banded ridge regression is hyperparameter Bayesian search [Nunez-Elizalde et al., 2019]. Hyperparameter Bayesian search [Mockus et al., 1978] is a method similar to hyperparameter random search. Both search methods compare a number of hyperparameter candidates and select the candidates with the lowest cross-validation error. The specificity of Bayesian search is that each candidate is selected conditionally

to the performances of previously tested candidates. The *Tikreg* Python package [Nunez-Elizalde et al., 2019] published previously by our group implements Bayesian search to solve banded ridge regression. Specifically, Tikreg implements a Bayesian search using trees of Parzen estimators [Bergstra et al., 2011] through the Hyperopt Python package [Bergstra et al., 2013].

A major limitation of hyperparameter Bayesian search is that it cannot optimize multiple losses simultaneously. For this reason, Tikreg cannot optimize a separate loss per voxel. Instead, Tikreg optimizes the *average* validation loss over all voxels. This approximation introduces a bias towards the preferred hyperparameters of the voxel majority. To mitigate this bias, Tikreg later selects the best hyperparameters per voxel out of all candidates explored by Bayesian search. However, this mitigation will only be successful if Bayesian search explores the hyperparameter space broadly and does not find its optimum quickly.

Another limitation of Tikreg's implementation of Bayesian search is that it does not use all computational tricks to solve the ridge regression subproblems. As described in Section 4.1, ridge regression can be solved efficiently by reusing computations for multiple regularization hyperparameters. To leverage this computational trick in random search (Section 4.5), hyperparameters are separated into $\gamma \in \Delta_{m-1}$ and $\mu > 0$. For each randomly-sampled candidate γ, a ridge regression is solved efficiently for a large number of μ. This factorization is key to explore hyperparameter space efficiently, but is not present in Tikreg's implementation.

A.6 Algorithms for weighted-kernel ridge regression

To solve multiple-kernel ridge regression, the hyperparameter gradient descent solver updates the kernel weights $\delta \in \mathbb{R}^m$ at each iteration. Then, at each iteration, the regression weights w must be optimized for the current (fixed) kernel weights $\delta \in \mathbb{R}^m$. We call this sub-problem *weighted-kernel ridge regression.* Specifically, for a *fixed* $\delta \in \mathbb{R}^m$, weighted-kernel ridge regression is defined as:

$$w^* = \underset{w}{\operatorname{argmin}} \left\| \sum_{i=1}^{m} e^{\delta_i} K_i w - y \right\|_2^2 + \sum_{i=1}^{m} e^{\delta_i} w^\top K_i w. \tag{10}$$

This section describes how to solve this sub-problem.

This problem has a closed formed solution $w^* = (K + I_n)^{-1} y$, where K is the kernel weighted sum $K = \sum_{i=1}^{m} e^{\delta_i} K_i$. However, if different voxels have different kernel weights δ, this closed-form solution is not efficient, as it requries computing K and inverting $(K + I_n)^{-1}$ for each different kernel weights δ. During hyperparameter gradient descent, a precise solution of the weighted-kernel ridge regression sub-problem is not necessary, and an approximation is sufficient [Pedregosa, 2016]. Thus, an approximate solution can be obtained with gradient descent (Algorithm 1), conjugate gradient descent (Algorithm 3), or even finite Neumann series (Algorithm 2). (Neumann series approximates $(K + I_n)^{-1} \approx f \sum_{j=1}^{k} (I_n - f(K + I_n))^j$, where the factor $0 < f < 1$ needs to be tuned to make the series converge.) All three algorithms are presented here for a single voxel, yet they can be straightforwardly extended to multiple voxels using matrices and tensors.

Interestingly, another sub-problem of hyperparameter gradient descent can be solved as a weighted-kernel ridge regression. During hyperparameter gradient descent, the gradient computation requires computing $\frac{\partial \mathcal{L}_{\text{val}}}{\partial w^*} \left(\frac{\partial^2 \mathcal{L}_{\text{train}}}{\partial w \partial w^\top} \right)^{-1}$; in multiple-kernel ridge regression, this term is equal to $K_{\text{val}}(K_{\text{val}} w - y_{\text{val}})(K_{\text{train}} + I_n)^{-1}$. This expression can be considered as the solution of equation (10), with $y = K_{\text{val}}(K_{\text{val}} w - y_{\text{val}})$. Therefore, the three approximate solvers of weighted-kernel ridge regression presented in this section can also be used to compute this element of the hyperparameter gradient computation.

In the example presented in Scetion 4.8, the ridge dual weights were updated with Algorithm 1 because Algorithm 2 was too coarse and Algorithm 3 led to oscillations in the convergence curves. For the hyperparameter gradient term, because the term did not require a precise approximation, all three algorithms (Algorithm 1, Algorithm 3, and Algorithm 2) were tested and compared.

Algorithm 1 Weighted-kernel ridge regression solver - Gradient descent

Input: target y, kernels $\{K_i\}_i$, fixed kernel weights δ, tolerance ε, initial dual weights w
compute $L(K_i)$ // Lipschitz constants
$\eta = 1/(\sum_{i=1}^{m} e^{\delta_i} L(K_i) + 1)$ // step size
$\Delta w = \infty$
while $\max(|\Delta w/w|) > \varepsilon$ **do**
    $\hat{y} = \sum_{i=1}^{m} e^{\delta_i}(K_i w)$ // prediction
    $\Delta w = -\eta(\hat{y} - y + w)$ // update
    $w = w + \Delta w$ // gradient step
end while
Output: weights w

Algorithm 2 Weighted-kernel ridge regression solver - Neumann series

Input: target y, kernels $\{K_i\}_i$, fixed kernel weights δ, number of terms k, factor f
$w = 0$ // sum accumulator
$p = y$ // product accumulator
for $j = 1$ **to** k **do**
    $Kp = \sum_{i=1}^{m} e^{\delta_i}(K_i p)$
    $p = p(1 - f) - fKp$
    $w = w + p$
end for
$w = fw$ // rescaling factor
Output: weights w

Algorithm 3 Weighted-kernel ridge regression solver - Conjugate gradient

Input: target y, kernels $\{K_i\}_i$, fixed kernel weights δ, tolerance ε, initial dual weights w
$\hat{y} = \sum_{i=1}^{m} e^{\delta_i}(K_i w)$ // prediction
$r_1 = -(\hat{y} - y + w)$ // negative gradient
$p = r_1$
$\Delta w = \infty$
for $k = 1$ **to** #iterations **do**
    $Kp = \sum_{i=1}^{m} e^{\delta_i}(K_i p)$
    $\alpha = r_k^\top r_k/(p^\top Kp)$
    $\Delta w = \alpha p$ // update
    $w = w + \Delta w$ // conjugate step
    if $\max(|\Delta w/w|) \leq \varepsilon$ **then**
        break for loop
    end if
    $r_{k+1} = r_k - \alpha Kp$
    $\beta = (r_{k+1}^\top r_{k+1})/(r_k^\top r_k)$
    $p = r_{k+1} + \beta p$
end for
Output: weights w

A.7 Algorithms for multiple-kernel ridge regression

This section describes algorithms to solve multiple-kernel ridge regression, optimizing $\delta \in \mathbb{R}^m$ over the validation loss:

$$\delta^* = \underset{\delta}{\operatorname{argmin}} \left\| \sum_{i=1}^{m} e^{\delta_i} K_{\text{val},i} w^*(\delta) - y_{\text{val}} \right\|_2^2, \tag{11}$$

where $w^*(\delta)$ is the solution of the weighted-kernel ridge regression (10). This problem can be either solved with hyperparameter random search (Algorithm 4) or with hyperparameter gradient descent (Algorithm 5).

Algorithm 4 Multiple-kernel ridge regression solver - Hyperparameter random search

Input: target Y, kernels $\{K_i\}_i$, Dirichlet's α, range of μ, cross-validation splits
for all voxels j **do**
  $\phi^*[j] = -\infty$ // initialization
  $\gamma^*[j] = \text{nan}$
  $\mu^*[j] = \text{nan}$
end for
for $\gamma \sim \text{Dirichlet}(\alpha)$ **do**
  $K = \sum_{i=1}^{m} \gamma_i K_i$ // kernel
  for all cross-validation splits s **do**
    $K_{\text{train}}, K_{\text{val}}, Y_{\text{train}}, Y_{\text{val}} = \text{split}_s(K, Y)$
    $UDU^\top = K_{\text{train}}$ // diagonalization
    for all regularizations μ **do**
      $M_\mu = K_{\text{val}} U (D + \mu_k I_n) U^\top$
      for all voxels j **do**
        $\hat{Y}_{\text{val}}[\mu, j] = M_\mu Y_{\text{train}}[j]$ // prediction
        $\phi[s, \mu, j] = ||Y_{\text{val}}[j] - \hat{Y}_{\text{val}}[\mu, j]||_2^2$
      end for
    end for
  end for
  $\bar{\phi}[\mu, j] = \text{mean}_s \phi[s, \mu, j]$ // mean over splits
  $\phi^*_\gamma[j] = \min_\mu \bar{\phi}[\mu, j]$ // min over mu
  $\mu^*_\gamma[j] = \text{argmin}_\mu \bar{\phi}[\mu, j]$
  for all voxels j **do**
    if $\phi^*[j] > \phi^*_\gamma[j]$ **then**
      $\phi^*[j] = \phi^*_\gamma[j]$ // update if improvement
      $\mu^*[j] = \mu^*_\gamma[j]$
      $\gamma^*[j] = \gamma$
    end if
  end for
end for
Output: $\gamma^*[j], \mu^*[j]$ for all voxels j

Algorithm 5 Multiple-kernel ridge regression solver - Hyperparameter gradient descent

Input: kernels $\{K_i\}_i$, initial δ, single target y
for k = 1 **to** #iterations **do**
  for all cross-validation splits s **do**
    $K_{\text{train}}, K_{\text{val}}, y_{\text{train}}, y_{\text{val}} = \text{split}_s(K, y)$

    // Update ridge dual weights w_s
    $w_s =$ Algorithm 1 $(y_{\text{train}}, \{K_{\text{train},i}\}_i, \delta, \varepsilon_k,\ w_s)$

    // Direct gradient
    $\hat{y}_{\text{val},i} = e^{\delta_i}(K_{\text{val},i} w_s)$ // partial prediction
    $r_{\text{val}} = (\sum_{i=1}^{m} \hat{y}_{\text{val},i} - y_{\text{val}})$ // residual
    $\frac{\partial \mathcal{L}_{\text{val}}}{\partial \delta_i} = r_{\text{val}} \hat{y}_{\text{val},i}$

    // Indirect gradient
    $\frac{\partial \mathcal{L}_{\text{val}}}{\partial w} = \sum_{i=1}^{m} e^{\delta_i}(K_{\text{val},i} r_{\text{val}})$
    $\omega_s =$ Algorithm 1, 2, or 3 $(\frac{\partial \mathcal{L}_{\text{val}}}{\partial w}, \{K_{\text{train},i}\}_i, \delta, \varepsilon_k, \omega_s)$
    $\hat{y}_{\text{train},i} = e^{\delta_i}(K_{\text{train},i} w_s)$ // partial prediction
    $\frac{\partial \mathcal{L}_{\text{val}}}{\partial w^*} \frac{\partial w^*}{\partial \delta} = \omega_s^\top \hat{y}_{\text{train},i}$

    // Full gradient
    $\frac{\partial \mathcal{L}^*_{\text{val}}}{\partial \delta_i}|_s = \frac{\partial \mathcal{L}_{\text{val}}}{\partial \delta_i} + \frac{\partial \mathcal{L}_{\text{val}}}{\partial w^*} \frac{\partial w^*}{\partial \delta}$

    // Step size
    $L_s =$ power iteration on the Hessian (9)
    $\eta_s = 1/L_s$
  end for
  $\delta = \delta - (\min_s \eta_s)(\text{mean}_s \frac{\partial \mathcal{L}^*_{\text{val}}}{\partial \delta_i}|_s)$
end for

References

[Andor et al., 2016] Andor, D., Alberti, C., Weiss, D., Severyn, A., Presta, A., Ganchev, K., Petrov, S., and Collins, M. (2016). Globally normalized transition-based neural networks. *arXiv preprint arXiv:1603.06042*.

[Araki and Lieb, 2002] Araki, H. and Lieb, E. H. (2002). Entropy inequalities. In *Inequalities*, pages 47–57. Springer.

[Bach, 2022] Bach, F. (2022). Information theory with kernel methods. *arXiv preprint arXiv:2202.08545*.

[Bach, 2008] Bach, F. R. (2008). Consistency of the group lasso and multiple kernel learning. *Journal of Machine Learning Research*, 9(Jun):1179–1225.

[Bach et al., 2004] Bach, F. R., Lanckriet, G. R., and Jordan, M. I. (2004). Multiple kernel learning, conic duality, and the SMO algorithm. In *International conference on machine learning*, page 6.

[Bach et al., 2005] Bach, F. R., Thibaux, R., and Jordan, M. I. (2005). Computing regularization paths for learning multiple kernels. In *Advances in neural information processing systems*, pages 73–80.

[Bartlett et al., 2020] Bartlett, P. L., Long, P. M., Lugosi, G., and Tsigler, A. (2020). Benign overfitting in linear regression. *Proceedings of the National Academy of Sciences*.

[Bergstra et al., 2013] Bergstra, J., Yamins, D., and Cox, D. D. (2013). Making a science of model search: Hyperparameter optimization in hundreds of dimensions for vision architectures. *Journal of machine learning research*.

[Bergstra et al., 2011] Bergstra, J. S., Bardenet, R., Bengio, Y., and Kégl, B. (2011). Algorithms for hyper-parameter optimization. In *Advances in neural information processing systems*, pages 2546–2554.

[Chapelle et al., 2002] Chapelle, O., Vapnik, V., Bousquet, O., and Mukherjee, S. (2002). Choosing multiple parameters for support vector machines. *Machine Learning*, 46(1):131–159.

[Foo et al., 2007] Foo, C.-s., Ng, A., et al. (2007). Efficient multiple hyperparameter learning for log- linear models. *Advances in neural information processing systems*, 20:377–384.

[Gönen and Alpaydın, 2011] Gönen, M. and Alpaydın, E. (2011). Multiple kernel learning algorithms. *The Journal of Machine Learning Research*, 12:2211–2268.

[Huth et al., 2016] Huth, A. G., De Heer, W. A., Griffiths, T. L., Theunissen, F. E., and Gallant, J. L. (2016). Natural speech reveals the semantic maps that tile human cerebral cortex. *Nature*, 532(7600):453–458.

[Huth et al., 2012] Huth, A. G., Nishimoto, S., Vu, A. T., and Gallant, J. L. (2012). A continuous semantic space describes the representation of thousands of object and action categories across the human brain. *Neuron*, 76(6):1210–1224.

[Keerthi et al., 2007] Keerthi, S. S., Sindhwani, V., and Chapelle, O. (2007). An efficient method for gradient-based adaptation of hyperparameters in SVM models. In *Advances in neural information processing systems*, pages 673–680.

[Kipper et al., 2006] Kipper, K., Korhonen, A., Ryant, N., and Palmer, M. (2006). Extending verbnet with novel verb classes. In *Proceedings of the Fifth International Conference on Language Resources and Evaluation (LREC'06)*.

[Klatzer and Pock, 2015] Klatzer, T. and Pock, T. (2015). Continuous hyper-parameter learning for support vector machines. In *Computer Vision Winter Workshop (CVWW)*, pages 39–47.

[Lanckriet et al., 2004] Lanckriet, G. R., Cristianini, N., Bartlett, P., Ghaoui, L. E., and Jordan, M. I. (2004). Learning the kernel matrix with semidefinite programming. *Journal of Machine learning research*, 5(Jan):27–72.

[Langeberg et al., 2019] Langeberg, P., Balda, E. R., Behboodi, A., and Mathar, R. (2019). On the effect of low-rank weights on adversarial robustness of neural networks. *arXiv preprint arXiv:1901.10371*.

[Larsen et al., 1996] Larsen, J., Hansen, L. K., Svarer, C., and Ohlsson, M. (1996). Design and regularization of neural networks: the optimal use of a validation set. In *Neural Networks for Signal Processing VI. Proceedings of the 1996 IEEE Signal Processing Society Workshop*, pages 62–71. IEEE.

[Lorraine et al., 2019] Lorraine, J., Vicol, P., and Duvenaud, D. (2019). Optimizing millions of hyperparameters by implicit differentiation. *arXiv preprint arXiv:1911.02590*.

[Mockus et al., 1978] Mockus, J., Tiesis, V., and Zilinskas, A. (1978). The application of bayesian methods for seeking the extremum. *Towards global optimization*, 2(117-129):2.

[Nishimoto et al., 2011] Nishimoto, S., Vu, A. T., Naselaris, T., Benjamini, Y., Yu, B., and Gallant, J. L. (2011). Reconstructing visual experiences from brain activity evoked by natural movies. *Current Biology*, 21(19):1641–1646.

[Nunez-Elizalde et al., 2018] Nunez-Elizalde, A., Deniz, F., Gao, J. S., and Gallant, J. L. (2018). Discovering brain representations across multiple feature spaces using brain activity. In *48th Annual Meeting of Society for Neuroscience (SfN), San Diego*.

[Nunez-Elizalde et al., 2019] Nunez-Elizalde, A. O., Huth, A. G., and Gallant, J. L. (2019). Voxelwise encoding models with non-spherical multivariate normal priors. *NeuroImage*, 197:482–492.

[Obozinski et al., 2011] Obozinski, G., Jacob, L., and Vert, J.-P. (2011). Group lasso with overlaps: the latent group lasso approach. *arXiv preprint arXiv:1110.0413*.

[Oord et al., 2016] Oord, A. v. d., Dieleman, S., Zen, H., Simonyan, K., Vinyals, O., Graves, A., Kalchbrenner, N., Senior, A., and Kavukcuoglu, K. (2016). Wavenet: A generative model for raw audio. *arXiv preprint arXiv:1609.03499*.

[Pan and Zhao, 2016] Pan, Q. and Zhao, Y. (2016). Integrative weighted group lasso and generalized local quadratic approximation. *Computational Statistics & Data Analysis*, 104:66–78.

[Pedregosa, 2016] Pedregosa, F. (2016). Hyperparameter optimization with approximate gradient. In *International Conference on Machine Learning*.

[Rakotomamonjy et al., 2008] Rakotomamonjy, A., Bach, F. R., Canu, S., and Grandvalet, Y. (2008). SimpleMKL. *Journal of Machine Learning Research*, 9(Nov):2491–2521.

[Roy and Vetterli, 2007] Roy, O. and Vetterli, M. (2007). The effective rank: A measure of effective dimensionality. In *2007 15th European Signal Processing Conference*, pages 606–610. IEEE.

[Saunders et al., 1998] Saunders, C., Gammerman, A., and Vovk, V. (1998). Ridge regression learning algorithm in dual variables. In *International conference on machine learning*.

[Tsuda et al., 2004] Tsuda, K., Uda, S., Kin, T., and Asai, K. (2004). Minimizing the cross validation error to mix kernel matrices of heterogeneous biological data. *Neural Processing Letters*, 19(1):63–72.

[Yuan and Lin, 2006] Yuan, M. and Lin, Y. (2006). Model selection and estimation in regression with grouped variables. *Journal of the Royal Statistical Society: Series B (Statistical Methodology)*, 68(1):49–67.
